# Improvement of AD-Census Algorithm Based on Stereo Vision

**DOI:** 10.3390/s22186933

**Published:** 2022-09-13

**Authors:** Yina Wang, Mengjiao Gu, Yufeng Zhu, Gang Chen, Zhaodong Xu, Yingqing Guo

**Affiliations:** 1 College of Mechanical and Electronic Engineering, Nanjing Forestry University, Nanjing 210037, China; 2 China-Pakistan Belt and Road Joint Laboratory on Smart Disaster Prevention of Major Infrastructures, Southeast University, Nanjing 210096, China

**Keywords:** stereo matching, AD-Census, obstacle detection, stereo vision

## Abstract

Problems such as low light, similar background colors, and noisy image acquisition often occur when collecting images of lunar surface obstacles. Given these problems, this study focuses on the AD-Census algorithm. In the original Census algorithm, in the bit string calculated with the central pixel point, the bit string will be affected by the noise that the central point is subjected to. The effect of noise results in errors and mismatching. We introduce an improved algorithm to calculate the average window pixel for solving the problem of being susceptible to the central pixel value and improve the accuracy of the algorithm. Experiments have proven that the object contour in the grayscale map of disparity obtained by the improved algorithm is more apparent, and the edge part of the image is significantly improved, which is more in line with the real scene. In addition, because the traditional Census algorithm matches the window size in a fixed rectangle, it is difficult to obtain a suitable window in the image range of different textures, affecting the timeliness of the algorithm. An improvement idea of area growth adaptive window matching is proposed. The improved Census algorithm is applied to the AD-Census algorithm. The results show that the improved AD-Census algorithm has been shown to have an average run time of 5.3% and better matching compared to the traditional AD-Census algorithm for all tested image sets. Finally, the improved algorithm is applied to the simulation environment, and the experimental results show that the obstacles in the image can be effectively detected. The improved algorithm has important practical application value and is important to improve the feasibility and reliability of obstacle detection in lunar exploration projects.

## 1. Introduction

Lunar exploration is a complex technical engineering field. It can promote the continuous development of high-tech technology, especially the development of aerospace technology, artificial intelligence, vibration control, mechanical analysis, and information technology [1,2,3,4,5]. Therefore, the exploration of the moon is of great significance. Lunar rovers are often used during lunar exploration. When the lunar rover is driving on the lunar surface, the rocks, potholes, and climbs on the lunar surface will bring difficulties to the autonomous cruising of the lunar rover. Therefore, the recognition effect of obstacles on the moon is good or bad, which directly affects the success or failure of the lunar rover’s autonomous cruise. Commonly used obstacle acquisition systems are lidar, the time-of-flight method (TOF), binocular cameras, etc. Lidar can produce high-precision depth information, but the device is expensive and consumes a lot of power. The TOF sensor can directly obtain the depth information of the image, but it is greatly affected by noise and it is expensive. The binocular vision device is small and cheap and has low power consumption, which can be used to detect obstacles encountered by the lunar rover when advancing and assist the lunar rover in a safer way forward in the exploration of the lunar surface.

In binocular stereo vision, two identical cameras at the same horizontal line are regarded as one person’s eyes. The two cameras maintain a certain distance, while the two-dimensional image of the same scene is collected. The disparity information of the scene is calculated by the acquisition of two images. Then, the disparity information is combined with the triangular geometric relationship to obtain the depth information of the scene. A complete binocular vision system consists of four main parts: camera calibration, image correction, stereo matching, and 3D reconstruction [6]. Stereo matching is the most important part, and it is also the most difficult. The goal of stereo matching is to find the corresponding pixel points from two images. The depth information of this point is calculated according to its disparity [7,8]. The accuracy of the disparity diagram obtained by stereo matching has a great influence on the subsequent three-dimensional reconstruction. Stereo matching has shown great application prospects and development potential in the industry. It has become one of the hot spots explored by researchers.

Stereo matching algorithms are constantly emerging, which promote the development of matching algorithms in the direction of high precision and real time. In the 1960s, L. Robert [9] of the Massachusetts Institute of Technology in the United States theoretically expounded the feasibility of stereo matching. This research was based on the acquired two-dimensional flat image data, through computer vision means, to process and expand to three-dimensional scenes. In the 1970s, Professor Marr of the Massachusetts Institute of Technology in the United States engaged in the study of machine vision theory based on the above theory and founded the computer vision theory [10]. It elaborates on all the processes of the computer, from the acquisition of the original two-dimensional image to the reconstruction of the three-dimensional object. The theory establishes that the transformation of the two-dimensional image to the three-dimensional scene needs to be realized through three steps and set the research direction of stereo matching. By the late 1980s, the active vision began to develop rapidly, and distance sensor fusion technology was introduced based on binocular vision, making stereo matching algorithms tend to mature. Barnard et al. [11] established a complete stereo matching system process based on Marr’s work, and proposed that in the stereo matching link, the most challenging and core link is stereo matching. Daniel Scharstein and Richard Szeliski [12] summarized the stereo matching algorithm into four steps: (1) matching cost calculation, (2) cost aggregation, (3) disparity calculation, and (4) disparity post-processing.

The traditional stereo matching algorithm can be divided into three categories, local matching, global stereo matching, and semi-local stereo matching. Local matching algorithms appeared earlier. For their simple calculation and high execution efficiency, they are easy to implement in hardware. The local matching algorithm is a good choice of solution in many cases and is the longest studied and most widely used algorithm in stereo matching. Zhou et al. [13] proposed a fast stereo matching algorithm based on an adaptive window. Yoon and Kweon et al. [14], Nalpantidis et al. [15], and Kowalczuk et al. [16] set different weight phases through a variety of matching algorithms to obtain support windows, assigned a weight to each pixel in the window, and then performed cost aggregation to obtain a disparity map. Peña et al. [17] and Keselman et al. [18] used the Census transformation algorithm in the cost calculation. It has a good matching effect in the smooth grayscale transformation area. Aiming at the problem that the traditional SAD similarity measure function easily causes amplitude distortion, Chai et al. [19] proposed a local stereo matching algorithm combining similarity measurement functions. The algorithm is effective and has better robustness to the conditions of light distortion and edge information. Wu et al. [20] presented a Bayesian inference-based multi-scale weighted voting framework, which utilized the rapidity of the local methods to construct a disparity space with scale information and made use of the complementarity of the disparity in different scales to find the best disparity distribution by Bayesian inference-based weighted voting. Liu et al. [21] proposed an efficient and effective matching cost measurement and an adaptive shape-guided filter-based matching cost aggregation method to improve the stereo matching performance for large texture-less regions. Compared with the traditional guided filter-based stereo matching method, the proposed method achieved a better matching result in texture-less regions. Building upon the existing local stereo matching algorithms, Zhang and Zhu [22] proposed a novel stereo matching algorithm that is based on a weighted guided filtering foundation, which could improve accuracy. Yuan et al. [23] proposed a novel fast gradient domain-guided image filter (F-GDGIF). It could achieve better edge-aware performance with a faster execution time and could generate more accuracy disparity maps with low computational cost compared to other GIF-based methods. Kong et al. [24] focused on matching cost computation and disparity refinement and proposed a gradient calculation method and a multistep refinement method based on ACR. Yang et al. [25] proposed an efficient local matching method based on an adaptive exponentially weighted moving average filter and simple linear iterative clustering segmentation algorithm. Qi et al. [26] proposed a stereo matching algorithm based on an improved adaptive support window.

With the rapid development of computer hardware, many researchers have further explored the three-dimensional matching algorithm and proposed a global stereo matching algorithm with a high computing degree by using the high-performance computing power of the computer. Roy et al. [27] proposed a global stereo matching algorithm based on the image-cutting method and used multiple iterations to remove the fringe phenomenon that appears in the general dynamic programming algorithm. Although the matching accuracy has been improved, the iterative process introduces a more complex amount of calculation, and the calculation speed is slower. Sun et al. [28] introduced Markov random airport into the stereo matching algorithm and proposed a stereo matching algorithm based on confidence propagation, which achieved good results. Vekslerp et al. [29] proposed a dynamic programming algorithm for tree structures, which further improved the efficiency of the algorithm. In view of the problem that the global algorithm based on the optimization of the energy function could improve the matching rate of the weak texture area in a small area, and the effect became inconspicuous in the large area of the weak texture area, Delong et al. [30] proposed an adaptive weight algorithm that could distinguish the weak texture area. Wang et al. [31] introduced bilateral filtering using the dynamic programming algorithm, which made the algorithm more real time. Yang et al. [32] proposed a confidence propagation matching algorithm based on control points, which effectively improved the accuracy of the algorithm. Li et al. [33] proposed a novel idea to improve both the efficiency and accuracy in global stereo matching for a long baseline to balance the matching efficiency and computing accuracy. Wang et al. [34] proposed a regional fuzzy binocular stereo matching algorithm based on global correlation coding, which could ensure the overall matching accuracy of left and right views and has a higher matching integrity.

Semi-local stereo matching is improved based on local and global stereo matching, with strong robustness, so semi-local stereo matching is also widely used. Hirschmüller et al. [35] proposed a semi-local stereo matching algorithm for SGM, using a calculation method based on mutual trust to calculate the matching cost between two graphs, using hierarchical computation to accelerate the implementation of the algorithm. The algorithm test effect took between 1 and 2 s, which verified that the SGM algorithm had good real-time performance. Guo et al. [36] improved the window of the Census transformation based on Census, which reduced the time complexity by comparing representative pixels while increasing the neighborhood window. Hamzah et al. [37] added gradient information to improve the initial matching accuracy and introduced iterative-guided filtering in the disparity optimization stage, further improving the accuracy of the disparity plot. Chai et al. [38] added the minimum spanning tree algorithm in the cost aggregation stage of the semi-global matching algorithm, which effectively improved the matching accuracy. Peng et al. [39] proposed an AGAP algorithm that added adaptive smoothing terms to the energy function, improving the disadvantages of one-dimensional scanline optimization in SGM algorithms. Rahnama et al. [40] proposed a novel, resource-efficient method inspired by MGM’s techniques for improving the depth quality, which could be implemented to run in real time on a low-power FPGA. Cambuim et al. [41] presented an FPGA-based stereo vision system based on SGM. This system calculated disparity maps by streaming, which were scalable to several resolutions and disparity ranges. As the matching accuracy of the traditional semi-global matching (SGM) algorithm is not satisfactory in illumination and weak texture areas, Li et al. [42] proposed a SGM algorithm based on multi-cost fusion. Bu et al. [43] introduced a local edge-aware filtering method to SGM to enhance the interaction of neighboring scanlines, since streak artifacts could be avoided. Li et al. [44] proposed a low-cost, high-precision method to identify and position charging ports based on SIFT and SGBM. Wei et al. [45] proposed a robust stereo matching system, RT-libSGM, working on the Xilinx field-programmable gate array (FPGA) platforms. The dedicated design of each module optimized the speed of the entire system while ensuring the flexibility of the system structure. Xu et al. [46] improved the existing second-order semi-global matching method and added the smoothness constraint of multiple angle directions to the matching cost to generate a more robust disparity map.

Although the current stereo matching algorithm is relatively mature, the images are affected by various external factors in the process of acquisition and there are still problems in how to improve the matching speed and accuracy. This paper focuses on the study of the AD-Census algorithm and improves it to improve the accuracy and speed of the algorithm. Aiming at the shortcomings of the traditional AD-Census algorithm, the accuracy of the algorithm is improved by replacing the pixel central value with the average of window pixels. To improve the timeliness of the algorithm, a method of replacing the traditional rectangular window of Census with an adaptive window is proposed.

## 2. Focused Problems

### 2.1. AD Algorithm

The main idea of the AD algorithm is to constantly compare the grayscale values of the two points in the left and right cameras. First, fix a point in the left camera, then traverse the points in the right camera, constantly comparing the difference in gray levels before them, and the difference in gray level is the cost of matching. The calculation formula is as follows:

(1) $$C_{AD}(x,y,d)=\left|I_{L}(x,y)-I_{R}(x+d,y)\right|$$

where *I_L_* and *I_R_* represent the grayscale values of the left and right images, respectively, *d* is the search range, and *x*, *y* are the pixel coordinates.

The AD algorithm is efficient and fast. However, it is easily disturbed by lighting and noise because the method only starts from a single grayscale angle.

### 2.2. Census Algorithm

Census transformation is a non-parametric area algorithm [47]. Its transformation is based on neighborhood grayscale while retaining the relative position information of neighborhood pixels. Therefore, the area matching algorithm based on Census transformation has good noise immunity for images with large brightness deviations and can obtain a good disparity map. However, the Census transformation relies on the pixels of the central point. If the central point pixels change considerably, it will affect the balance of the surrounding neighborhood pixels. Census first determines the window with the pixel point *p* as the central point and compares the gray value of the field with the gray value *p* of the central pixel point. If the pixel *p* is larger than the surrounding pixels, the larger pixel is recorded as 0. Otherwise, it is recorded as 1. Finally, the resulting transformation codes of the pixel points can be derived by concatenating them according to the bits. According to the above process, it can be described by the following formula:

(2) $$C_{T}\left(p\right)=\underset{q\in N}{⊗}\sigma \left(I\left(p\right),I\left(q\right)\right)$$

where σ(I(p),I(q)) is the comparison function, *p* is the central pixel, and *q* is the domain pixel in the window centralized on *p*. *I*(*p*), *I*(*q*) are the grayscale values of pixel points *p* and *q*, respectively. When the pixel point p is larger than the surrounding pixels, the larger pixel is recorded as 0, otherwise it is recorded as 1. The calculation formula is as follows:

(3) $$\sigma (I(p),I(q))=\{\begin{array}{c} 0\;\;I(p)\leq I(q)\\ 1\;\;I(p)>I(q) \end{array}$$

After setting in the fixed window, arrange 1 or 0 in all windows into a bit string in order. The bit strings of pixels in the two images are compared and the matching cost can be obtained by using the Hamming distance similarity measure, as shown below:

(4) $$h(p,d)=Hamming\left(C_{TL}(p),C_{TR}(p,d)\right)$$

**Figure 1:**
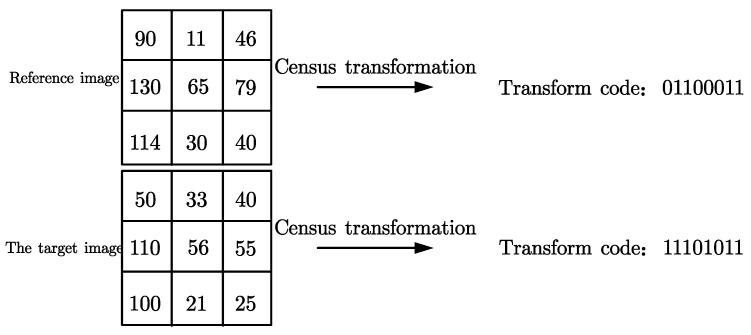
Census transformation process.

**Figure 2:**
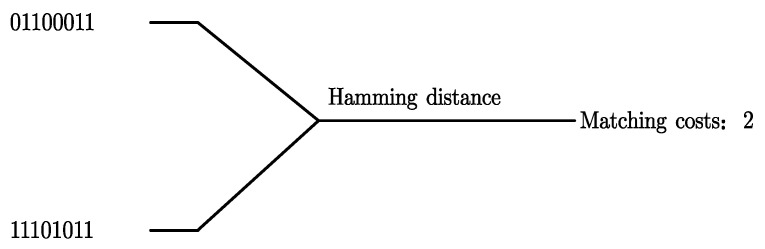
Hamming distance similarity detection.

We take a 3 × 3 template as an example. Figure 1 shows the transformation process of Census. The transformation codes can be calculated as 01100011 and 11101011 using the formula. The two transformation codes are XORed and summed, and the matching cost of the two points is 2, as shown in Figure 2.

Although the Census transform can well-correct the grayscale deviation caused by uneven illumination, the Census transform only uses a 0/1-bit byte string to represent the grayscale difference between two pixels, making the feature descriptor too large. The matching cost obtained by Census transformation cannot reflect the similarity of images well when the grayscale changes in adjacent windows are sharp or the same repeated area exists.

In a word, AD transform is greatly affected by illumination. In contrast, Census transform can maintain good adaptability to light changes and has a better correction effect on the gray value changes of pixel points caused by uneven illumination. Therefore, the improved AD-Census transform algorithm is proposed to better use AD and Census transform.

## 3. Improved AD-Census Algorithm

AD transform and Census transform have different scales, so it is necessary to normalize the results of the two, processing them into the same result range. The AD-Census algorithm uses the exponential form of [0,1] to control the parameter changes and controls the values obtained by AD transformation and Census transformation between 0 and 1, to prevent one of them from changing too much and affecting the output result. The specific formula is as follows:

(5) $$\rho (c,\lambda )=1-\exp\left(-\frac{c}{\lambda }\right)$$

where *c* is the surrogate value and λ is the control parameter. Any surrogate value can be normalized to [0, 1].

The final cost formula is obtained, which allows to control the AD-Census normalization result between [0, 2].

(6) $$C(p,d)=p\left(C_{census}(p,d),\lambda_{census}\right)+p\left(C_{AD}(p,d),\lambda_{AD}\right)$$

In this paper, the improved Census algorithm is applied to the AD-Census algorithm.

### 3.1. Noise Reduction

The Census algorithm relies too much on the central pixel point. In the bit string calculated with the central pixel point, the bit string will be affected by the noise that the central point is subjected to, which results in errors and mismatching. Therefore, this improvement method first calculates the average value of the window pixels, and then uses it to replace the central pixel.

**Figure 3:**
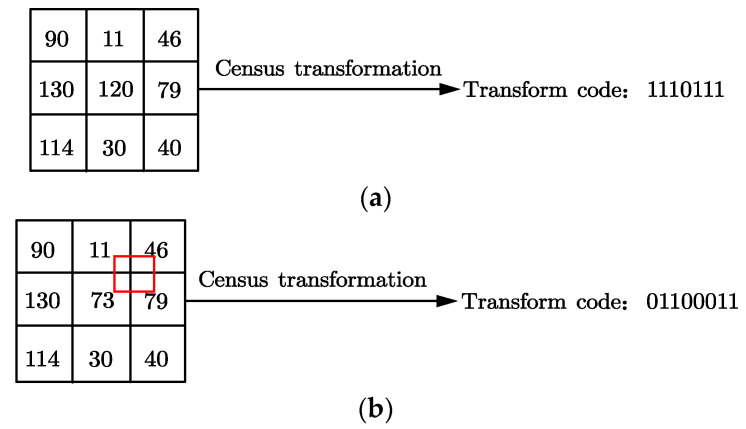
Census transformation comparison diagram. (**a**) The central pixel is not discriminated by interference. (**b**) The central pixel is interfered with to discriminate.

As shown in Figure 3, under the 3 × 3 window of conventional Census, the central pixel point becomes 120 due to noise. The bit string after Census transformation is 1110111 at this time. After the mean value is calculated, the central pixel is replaced by 73 with 120, and the bit string 0110011 is calculated.

Noise interference can be successfully reduced by the average operation. Therefore, it is by replacing the average pixel value with the central pixel value that the accuracy and matching effect can be effectively improved.

### 3.2. Adaptive Window

The method of replacing the central pixel point with the mean value can enhance the matching accuracy of the algorithm and reduce the dependence of the Census transform on the central pixel point. However, the computation will greatly increase the complexity of the algorithm. It is also not conducive to the real-time performance of the algorithm. Therefore, the operation rate of the algorithm also needs to be improved.

Since the size of the matching window of the traditional Census algorithm is a fixed rectangle, it is difficult to obtain a suitable window in the image range of different textures, which affects the timeliness of the algorithm. Based on this, an improved idea of the regional growth adaptive window is proposed. The specific algorithm steps are shown in the following equation [48]:

(7) $$\{\begin{array}{l} D_{c}\left(p_{1},p\right)<\tau_{1}\;and\;D_{c}\left(p_{1},p+(1,0)\right)<\tau_{1}\\ D_{s}\left(p_{1},p\right)<L_{1}\\ D_{c}\left(p_{1},p\right)<\tau_{2}\;if\;L_{2}<D_{s}\left(p_{1},p\right)<L_{1} \end{array}$$

where *p* is the point in the image to be matched. The growth window is set from the coordinates of point *p* to the positive and negative directions of the x-axis, and the growing arm length is determined by setting the threshold. When the threshold condition is met, the newly added point *p*_1_ is included in the matching window. If the threshold condition is not met, the growth will be stopped. The threshold of the adaptive window is set to grayscale difference, which is different from the color similarity and luminance difference set in the cross-based cost aggregation (CBCA) [49] and the original AD-Census algorithm. The formed area is the adaptive window. There are three specific cases:

① When the difference in the gray level between pixel *p* and the new growth point *p*_1_ is less than the set threshold $\tau_{1}\;$, and the difference in the gray level between the next pixel and the new growth point *p*_1_ is also less than the set threshold $\tau_{1}\;$, the growth in that direction is stopped.

② When the arm length, $D_{s}\left(p_{1},p\right)$, between the new growth points *p*_1_ and *p* is less than the set arm length L_1_, the growth in this direction is stopped at this time.

③ When the arm length is less than the set arm length L_1_ but greater than the set arm length L_2_, and the grayscale difference between *p*_1_ and *p* is less than a smaller threshold value $\tau_{2}$, the growth is stopped.

Condition ① is better to find the area with a small gray value difference and limit the difference to grow to the next point, so that the points with similar gray values can be better found. Conditions ② and ③ are to find as many similar grayscale points as possible. However, for arm lengths larger than a smaller arm length, the grayscale value of the pixel is required to be smaller than a smaller grayscale difference threshold to include the pixel in the matching window.

The improved algorithm can obtain adaptive windows of different shapes and sizes compared to the traditional Census fixed rectangular window. The grayscale values within the window have better continuity, which is conducive to improving the matching accuracy.

### 3.3. Improved AD-Census Algorithm

**Figure 4:**
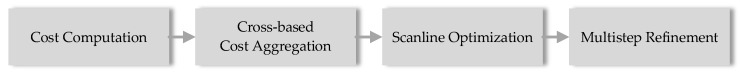
The flow chart of the improved AD-Census algorithm.

The improved Census algorithm is applied to the AD-Census algorithm, and the flow of the improved AD-Census algorithm is as follows:

(1) Cost computation. The similarity between the left and right images is calculated and then evaluated. The AD algorithm and the Census algorithm are used to calculate the matching cost, respectively. The results of the two algorithms are fused to form the AD-Census cost. In the cost computation with the Census algorithm, the central value of each pixel point is replaced with the average value to achieve noise reduction and improve the matching accuracy.
(2) Cross-based cost aggregation. In this paper, we use the same cost aggregation method, CBCA, as the original AD-Census algorithm. In the cost aggregation, two iterations are used, which differs from the four iterations in the original algorithm. The direction of iteration is also different from CBCA. The first iteration grows horizontally and then grows vertically in the window, and the second iteration is the exact opposite. The smaller of the two is taken as the cost aggregation value, which is also different from the final aggregated generation value in the original algorithm. In this way, the mismatching rate in the disparity discontinuity region can be effectively reduced.
(3) Scanline optimization. After the cost aggregation, the most suitable disparity value is selected from the disparity map.
(4) Multistep refinement. The accuracy of the algorithm can be improved by detecting and eliminating errors that arise due to errors in the first three steps.
(5) The flow chart of the improved AD-Census algorithm is as shown in Figure 4.

Therefore, the improved algorithm can reduce noise and improve the accuracy of the algorithm by replacing the pixel central value with a window pixel mean. A kind of rectangular window is proposed to replace the traditional rectangular window of Census with an adaptive window, which can improve the timeliness of the algorithm.

## 4. Results and Discussion

To verify the effect of the improved algorithm, we tested the four sets of standard test images provided by the international stereo matching evaluation platform, including Cones, Teddy, Tsukuba, and Venus. They were tested through the OpenCV platform. The matching rate can reflect the algorithm operation rate and the optimization of the program, so we compared the algorithm running times, as shown in Table 1. The data show that for all tested image sets, the improved AD-Census algorithm reduced the run time by an average of 5.3% compared to the traditional AD-Census algorithm and matched better.

**Figure 5:**
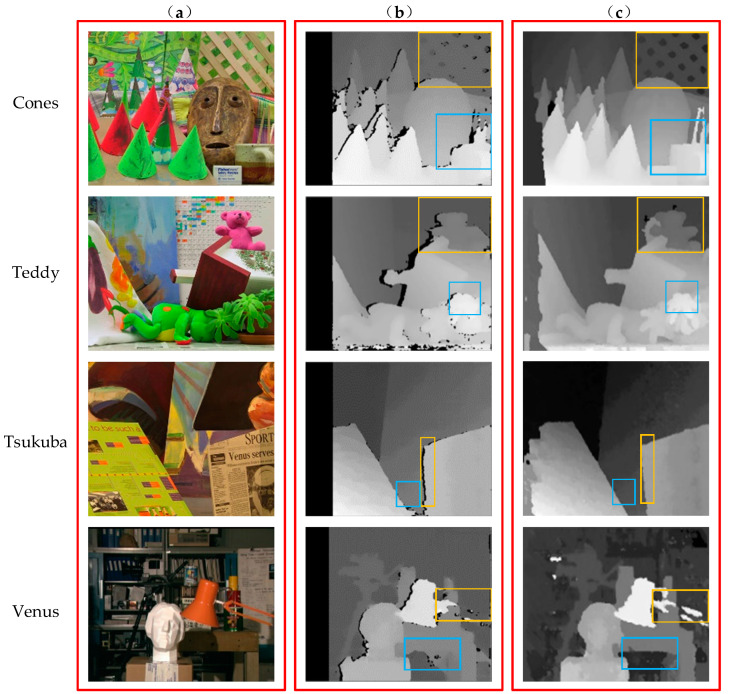
Comparison of algorithm results: (**a**) the original diagram corresponding to the four groups of images of Cones, Teddy, Tsukuba, and Venus, respectively, (**b**) the disparity map corresponding to the traditional AD-Census algorithm, corresponding to the four groups of images, and (**c**) the disparity map of the improved AD-Census algorithm corresponding to the four groups of images.

The comparison chart before and after the improvement of the AD-Census algorithm is shown in Figure 5. It can be seen that compared with the traditional AD-Census algorithm, the improved AD-Census algorithm obtained a sharper outline of the object in the disparity grayscale map after adding the window mean replacement window central value and combining the adaptive window. As shown in the rectangular boxed portion of the figure, the edge part of the image was significantly improved, which is more suitable for the actual scene. In summary, the running time of the improved AD-Census algorithm was effectively reduced and the matching accuracy was also improved.

In this paper, we also compared the objective performance indicators of the two algorithms, as shown in Table 2, Table 3 and Table 4. As can be seen in Table 2, the mean square error (MSE) of the traditional AD-Census algorithm was much larger than that of the improved AD-Census algorithm. The result indicates that the quality of the disparity maps obtained by the improved AD-Census algorithm was better than that of the traditional AD-Census algorithm. In Table 3, the peak signal-to-noise ratio (PNSR) of the traditional AD-Census algorithm was significantly lower than that of the improved AD-Census algorithm. This indicates that the noise of the disparity maps obtained by the improved AD-Census algorithm was reduced and confirms the noise reduction effect of the improved algorithm. From Table 4, it can be seen that the structure similarity index measure (SSIM) of the improved AD-Census algorithm was higher than that of the traditional AD-Census algorithm. This indicates that the disparity map obtained by the improved algorithm fits better with the original map.

**Figure 6:**
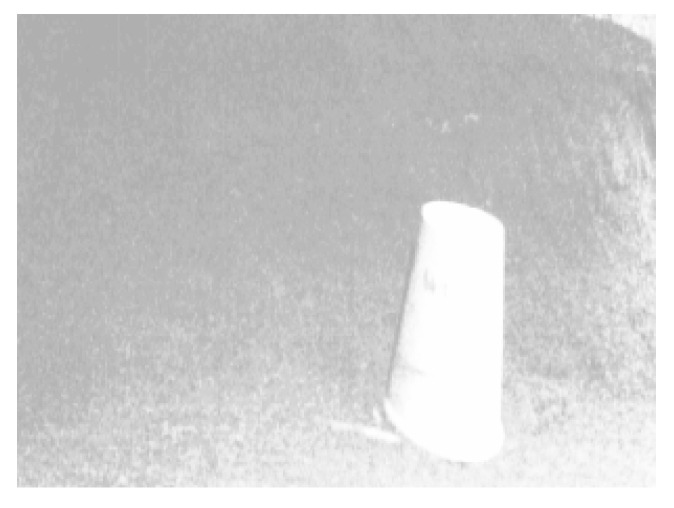
Disparity map in a simulated environment.

**Figure 7:**
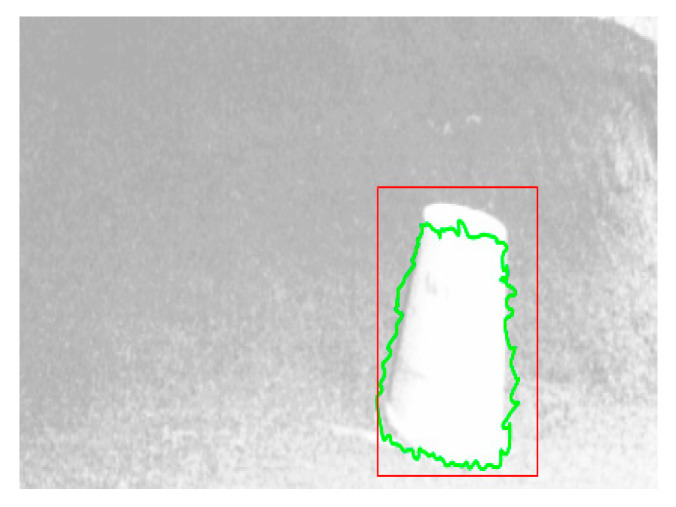
Obstacle detection diagram.

The improved algorithm was applied to the real scene, and we obtained the disparity plot shown in Figure 6. After taking the disparity diagram of the picture in the simulated environment obtained, the disparity map was pre-processed, and underwent edge detection, morphological processing, and obstacle extraction. Finally, the location of the obstacle was identified on the original map. Figure 7 shows the resulting obstacle recognition rendering. It can be clearly and intuitively seen that the obstacles in the picture were effectively detected.

## 5. Conclusions

In summary, we focused on studying the AD-Census algorithm and proposed a technique to improve it. We proposed a method to calculate the average window pixel instead of the window pixel central value to improve the accuracy of the algorithm. An improvement idea of area growth adaptive window matching was proposed to improve the timeliness of the algorithm. The experimental results proved that the object outline in the disparity grayscale map obtained by the improved algorithm was more apparent, and the edge part of the image was significantly improved, which was more in line with the real scene. For all tested image sets, the improved AD-Census algorithm reduced the run time by 5.3% compared to the traditional AD-Census algorithm and matched better. It was found that the obstacle in the graph could be well-identified for the improved algorithm for obstacle detection. The experiment results showed that the proposed method had good accuracy and speed. It can be applied to more complex backgrounds and difficult scenes with dim light. The improved algorithm can improve the feasibility and reliability of obstacle detection in the lunar exploration project and has significant practical application value. Although the improved AD-Census has improved the speed, it has not yet met the effect of high real-time performance. Therefore, the algorithm speed needs to be further improved in the future study and different obstacles need to be classified and identified.

## Figures and Tables

**Table 1:** Comparison of algorithms’ running times.

	Image Set/s			
Algorithm	Cones	Teddy	Tsukuba	Venus
Traditional AD-Census algorithm	3.149	2.854	1.912	2.755
Improved AD-Census algorithm	3.024	2.578	1.802	2.736

**Table 2:** Comparison of algorithms’ MSE.

	MSE			
Algorithm	Cones	Teddy	Tsukuba	Venus
Traditional AD-Census algorithm	7989.979	10,155.730	4812.042	4309.310
Improved AD-Census algorithm	6403.497	7030.245	2929.029	3446.573

**Table 3:** Comparison of algorithms’ PSNR.

	PSNR/dB			
Algorithm	Cones	Teddy	Tsukuba	Venus
Traditional AD-Census algorithm	27.755	27.874	27.888	27.802
Improved AD-Census algorithm	28.080	28.005	27.979	27.965

**Table 4:** Comparison of algorithms’ SSIM.

	SSIM			
Algorithm	Cones	Teddy	Tsukuba	Venus
Traditional AD-Census algorithm	0.138	0.231	0.239	0.173
Improved AD-Census algorithm	0.240	0.324	0.348	0.241

## Data Availability

Publicly available datasets were analyzed in this study. This data can be found here: [vision.middlebury.edu].
